# Poly[[diaqua­{*N*-[1-(3-pyrid­yl)ethyl­idene]-4*H*-1,2,4-triazol-4-amine}zinc(II)] bis­(perchlorate)]

**DOI:** 10.1107/S1600536809013130

**Published:** 2009-04-10

**Authors:** Xiaodan Sun, Xianhua He, Wei Wang, Donghua Miao, Qiaozhen Sun

**Affiliations:** a Department of Materials Chemistry, School of Materials Science and Engineering, Key Laboratory of Non-ferrous Metals of the Ministry of Education, Central South University, Changsha 410083, People’s Republic of China

## Abstract

In the title compound, {[Zn(C_9_H_9_N_5_)_2_(H_2_O)_2_](ClO_4_)_2_}_*n*_, the Zn^II^ ion lies on an inversion center and is coordinated by two triazolyl N atoms and two pyridyl N atoms from four symmetry-related *N*-1-(3-pyrid­yl)ethyl­idene-4*H*-1,2,4-triazol-4-amine (*L*) ligands and two O atoms from coordinated water mol­ecules in a slightly distorted octa­hedral environment. Each *L* ligand bridges symmetry-related Zn^II^ ions, forming a two-dimensional layer with a (4,4) grid. In the crystal structure, inter­molecular O—H⋯O hydrogen bonds connect perchlorate counter-anions to the layers.

## Related literature

For the structures of triazole complexes, see: Wang *et al.* (2006 ▶, 2007 ▶); Drabent *et al.* (2003 ▶, 2004 ▶, 2008 ▶); Sun *et al.* (2009*a* ▶,*b* ▶); Yi *et al.* (2004 ▶). For general background information, see: Beckmann & Brooker (2003 ▶); Ding *et al.* (2007 ▶); Haasnoot (2000 ▶); Klingele & Brooker (2003 ▶); Zhai *et al.* (2006 ▶). For the (4,4) topology, see: Batten & Robson (1998 ▶).
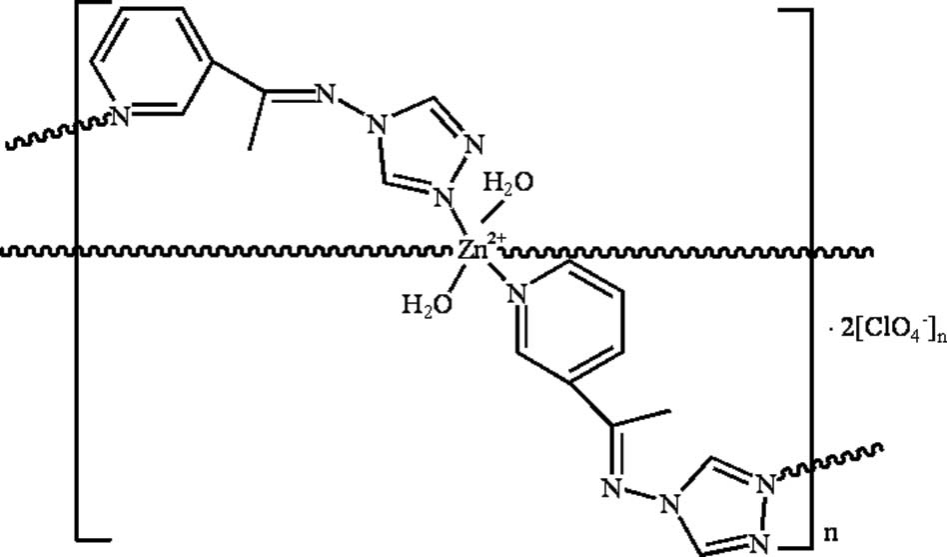


## Experimental

### 

#### Crystal data

[Zn(C_9_H_9_N_5_)_2_(H_2_O)_2_](ClO_4_)_2_
*M* *_r_* = 674.75
Monoclinic, 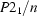

*a* = 7.4929 (9) Å
*b* = 10.0963 (12) Å
*c* = 17.149 (2) Å
β = 94.887 (2)°
*V* = 1292.6 (3) Å^3^
*Z* = 2
Mo *K*α radiation
μ = 1.23 mm^−1^
*T* = 293 K
0.38 × 0.30 × 0.30 mm

#### Data collection

Bruker SMART CCD diffractometer
Absorption correction: multi-scan (*SADABS*; Bruker, 2000 ▶) *T*_min_ = 0.647, *T*_max_ = 0.697
6344 measured reflections
2266 independent reflections
1856 reflections with *I* > 2σ(*I*)
*R*_int_ = 0.053

#### Refinement

*R*[*F* ^2^ > 2σ(*F* ^2^)] = 0.044
*wR*(*F* ^2^) = 0.130
*S* = 1.05
2266 reflections
187 parameters
H-atom parameters constrained
Δρ_max_ = 0.34 e Å^−3^
Δρ_min_ = −0.35 e Å^−3^

### 

Data collection: *SMART* (Bruker, 2000 ▶); cell refinement: *SAINT* (Bruker, 2000 ▶); data reduction: *SAINT*; program(s) used to solve structure: *SHELXTL* (Sheldrick, 2008 ▶); program(s) used to refine structure: *SHELXTL*; molecular graphics: *SHELXTL*, *DIAMOND* (Brandenburg & Putz, 1999 ▶) and *OLEX* (Dolomanov *et al.*, 2003 ▶); software used to prepare material for publication: *SHELXTL*.

## Supplementary Material

Crystal structure: contains datablocks global, I. DOI: 10.1107/S1600536809013130/lh2801sup1.cif

Structure factors: contains datablocks I. DOI: 10.1107/S1600536809013130/lh2801Isup2.hkl

Additional supplementary materials:  crystallographic information; 3D view; checkCIF report

## Figures and Tables

**Table 1:** Hydrogen-bond geometry (Å, °)

*D*—H⋯*A*	*D*—H	H⋯*A*	*D*⋯*A*	*D*—H⋯*A*
O1*W*—H1*WA*⋯N2	0.85	2.20	2.814 (4)	130
O1*W*—H1*WB*⋯O4^i^	0.85	2.22	2.993 (5)	151
O1*W*—H1*WB*⋯O3^i^	0.85	2.26	3.003 (5)	147

Symmetry code: (i) 
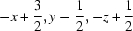
.

**Fig. 1.**
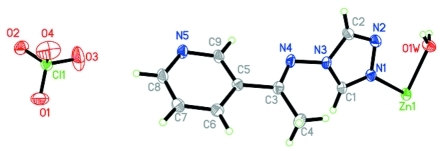
The asymmetric unit of the title compound showing the atom-numbering scheme. Displacement ellipsoids are drawn at the 30% probability level.

**Fig. 2.**
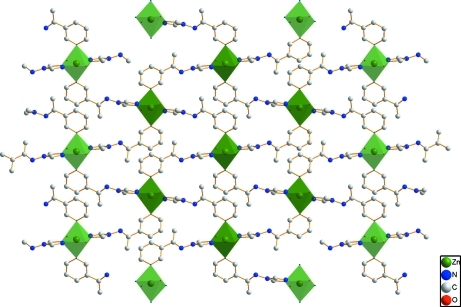
Part of the crystal structure showing the (4, 4) topology and the slightly distorted octahedal configuration for ZnII ions.

**Fig. 3.**
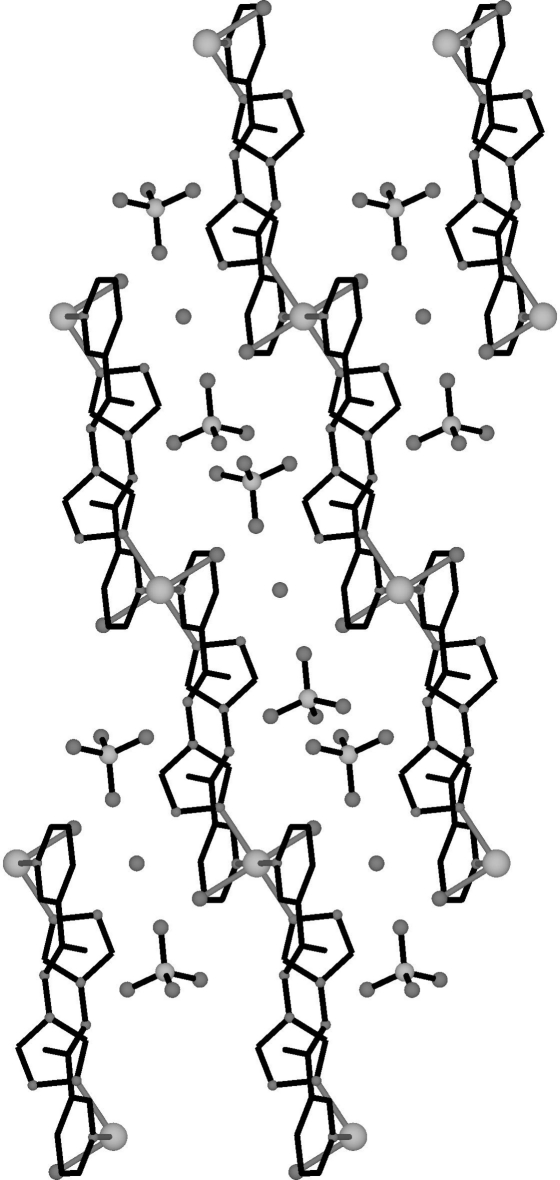
Part of the crystal structure viewed perpendicular to the (010) plane to show how ClO4- anions and coordinated water ligands occupy the layers. H atoms have been omitted for clarity.

## supplementary crystallographic information

### Comment

1,2,4-Triazoles and their derivatives can coordinate with metal ions using two bridging adjacent nitrogen atoms via the 1, 2 or 4-positioned N atoms, exhibiting unique magnetic properties. Recently, a variety of such coordination compounds with various structures and different chemical properties have been reported (Beckmann & Brooker, 2003; Ding *et al.*, 2007; Haasnoot, 2000; Klingele & Brooker, 2003; Zhai *et al.*, 2006). Relatively speaking, the crystal structures of only a few compounds based on 4-amido-1,2,4-triazoles Schiff base ligands have been studied e.g. [Ag_4_(µ2-*L*)_6_(CH_3_CN)_2_] (AsF_6_)_4_.2H_2_O [where *L*=4-salicylideneamino- 1,2,4-triazole] (Wang *et al.*, 2006) and a series of one-dimensional linear chain polymers {[Cu(µ-OH)(µ-RPhtrz)] [(H_2_O)*X*]}_n_ (where *R*=Cl, Br; HPhtrz= *N*-[(*E*)-phenylmethylidene-4*H*-1,2,4-triazol-4- amine]; *X*=BF_4_^-^, NO_3_^-^) (Drabent *et al.*, 2008). However, the most common structure type is dimeric with *M*_2_*L*_4_ [where *M*=Cu(I), Ag(I)] in which two ligands coordinate with metal ion in monodentate fashion and two in bidentate mode (Drabent *et al.*, 2003;2004; Wang *et al.*, 2007).

As part of our on-going work (Sun *et al.*, 2009*a*,*b*), we have synthesized *N*-[1-(3-pyridyl)ethylidene]-4*H*-1,2,4-triazol- 4-amine. Unlike above Schiff base ligands containing 1,2,4-triazole, it is a rigid angular multifunctional ligand containing one pyridine and one triazole group, which are both strong coordination donors to metal centers. Therefore, it was expected that the pyridyl N atom would coordinate with metal ions creating a structure with a novel topology. Herein we present the crystal structure of the title two-dimensional layer compound with a (4,4) grid (Batten & Robson, 1998).

The asymmetric unit of the title compound  is shown in Fig. 1. Each Zn^II^ ion is in a slightly distorted octahedral coordination environment with the equatorial sites occupied by two triazolyl N atoms of symmetry related ligands (*L*) and two symmetry related water molecules. The axial sites are occupied by two pyridyl N atoms from two symmetry related ligands (*L*). Unlike the N1, N2 coordination mode reported previously (Drabent *et al.*, 2003,2004,2008; Sun *et al.*, 2009*a*,*b*; Wang *et al.*, 2006 and 2007; Yi *et al.*, 2004), each ligand in the title compound bridges two Zn^II^ ions, forming a two-dimensional sheet with a (4, 4) topology (Fig. 2). Fig. 3 shows how ClO_4_^-^ anions and coordinated water molecules occupy the spaces between neighbouring layers.

### Experimental

Preparation of ligand *L*: An ethanolic solution (20 ml) of 3-acetylpyridine (1.21 g, 10 mmol) was added to a warm ethanolic solution (10 ml) of 4-amino- 1,2,4-triazole (0.84 g, 10 mmol) and the resulting solution was refluxed for four hours. The reaction mixture was then cooled to room temperature. Upon standing overnight the resultant pale yellow solid was filtered off, washed with diethyl ether and dried under vacuum. Yield: 80%. Elemental analyses calcd (%): C, 57.7; H, 4.8; N, 37.4. Found: C, 57.6; H, 4.8; N, 37.4. 1H NMR (500 MHz, DMSO, 298 K): 9.14 (d, 1H), 8.80 (s, 2H), 8.77 (d, 1H), 8.35–8.36 (d, 1H), 7.57–7.59 (m, 1H), 2.44 (s, 3H).

Preparation of the title compound: The ligand *L* (0.1 mmol, 0.019 g) and Zn(ClO_4_)_2_.6H_2_O (0.1 mmol, 0.037 g) were mixed in acetonitrile and methanol. After stirring at room temperature for one hour, the colourless solution was filtered and evaporated at room temperature. A few days later the block crystals were obtained. Elemental analyses calcd (%) for Zn_0.5_C_9_H_11_ClN_5_O_5_: C, 30.8; H, 4.6; N, 20.0. Found: C, 30.7; H, 4.5; N, 20.0. IR (KBr pellets, λ, cm^-1^): 3384*m*, 3124*m*, 1627*m*, 1588w, 1523*m*, 1477w, 1420w, 1369w, 1291*m*, 1184*m*, 1088vs, 1010*m*, 888w, 826w, 698*m*, 626 s, 489w, 435w.

### Refinement

H atoms were placed calculated positions C-H = 0.93-0.96Å; O-H = 0.85Å and included in a riding-model approximation with U_iso_(H) = 1.2U_eq_(C,O).

### Crystal data

**Table:**

[Zn(C_9_H_9_N_5_)_2_(H_2_O)_2_](ClO_4_)_2_	*F*(000) = 688
*M**_r_* = 674.75	*D*_x_ = 1.734 Mg m^−^^3^
Monoclinic, *P*2_1_/*n*	Mo *K*α radiation, λ = 0.71073 Å
Hall symbol: -P 2yn	Cell parameters from 2652 reflections
*a* = 7.4929 (9) Å	θ = 2.4–27.8°
*b* = 10.0963 (12) Å	µ = 1.23 mm^−^^1^
*c* = 17.149 (2) Å	*T* = 293 K
β = 94.887 (2)°	Block, colourless
*V* = 1292.6 (3) Å^3^	0.38 × 0.30 × 0.30 mm
*Z* = 2	

### Data collection

**Table:**

Bruker SMART CCD diffractometer	2266 independent reflections
Radiation source: fine-focus sealed tube	1856 reflections with *I* > 2σ(*I*)
graphite	*R*_int_ = 0.053
φ and ω scans	θ_max_ = 25.0°, θ_min_ = 2.3°
Absorption correction: multi-scan (*SADABS*; Bruker, 2000)	*h* = −8→8
*T*_min_ = 0.647, *T*_max_ = 0.697	*k* = −11→11
6344 measured reflections	*l* = −20→18

### Refinement

**Table:**

Refinement on *F*^2^	Primary atom site location: structure-invariant direct methods
Least-squares matrix: full	Secondary atom site location: difference Fourier map
*R*[*F*^2^ > 2σ(*F*^2^)] = 0.044	Hydrogen site location: inferred from neighbouring sites
*wR*(*F*^2^) = 0.130	H-atom parameters constrained
*S* = 1.05	*w* = 1/[σ^2^(*F*_o_^2^) + (0.0859*P*)^2^ + 0.0827*P*] where *P* = (*F*_o_^2^ + 2*F*_c_^2^)/3
2266 reflections	(Δ/σ)_max_ < 0.001
187 parameters	Δρ_max_ = 0.34 e Å^−^^3^
0 restraints	Δρ_min_ = −0.35 e Å^−^^3^

### Special details

**Table:**

Geometry. All e.s.d.'s (except the e.s.d. in the dihedral angle between two l.s. planes) are estimated using the full covariance matrix. The cell e.s.d.'s are taken into account individually in the estimation of e.s.d.'s in distances, angles and torsion angles; correlations between e.s.d.'s in cell parameters are only used when they are defined by crystal symmetry. An approximate (isotropic) treatment of cell e.s.d.'s is used for estimating e.s.d.'s involving l.s. planes.
Refinement. Refinement of *F*^2^ against ALL reflections. The weighted *R*-factor *wR* and goodness of fit *S* are based on *F*^2^, conventional *R*-factors *R* are based on *F*, with *F* set to zero for negative *F*^2^. The threshold expression of *F*^2^ > σ(*F*^2^) is used only for calculating *R*-factors(gt) *etc*. and is not relevant to the choice of reflections for refinement. *R*-factors based on *F*^2^ are statistically about twice as large as those based on *F*, and *R*- factors based on ALL data will be even larger.

### Fractional atomic coordinates and isotropic or equivalent isotropic displacement parameters (Å^2^)

**Table:**

	*x*	*y*	*z*	*U*_iso_*/*U*_eq_	
Zn1	0.5000	0.0000	0.0000	0.0318 (2)	
N1	0.6739 (4)	0.0235 (3)	0.10252 (15)	0.0313 (6)	
N2	0.8545 (4)	0.0414 (3)	0.09414 (16)	0.0388 (7)	
N3	0.8080 (3)	0.0549 (3)	0.21762 (14)	0.0295 (6)	
N4	0.8448 (4)	0.0976 (3)	0.29546 (14)	0.0339 (6)	
N5	0.9165 (4)	0.2865 (3)	0.50082 (15)	0.0346 (6)	
C1	0.6489 (4)	0.0320 (3)	0.17647 (18)	0.0311 (7)	
H1B	0.5392	0.0237	0.1976	0.037*	
C2	0.9296 (4)	0.0616 (4)	0.16374 (18)	0.0375 (8)	
H2B	1.0511	0.0784	0.1753	0.045*	
C3	0.7815 (4)	0.0301 (3)	0.34965 (19)	0.0305 (7)	
C4	0.6845 (5)	−0.0988 (3)	0.3412 (2)	0.0433 (9)	
H4B	0.6757	−0.1257	0.2873	0.065*	
H4C	0.5665	−0.0889	0.3582	0.065*	
H4D	0.7489	−0.1648	0.3726	0.065*	
C5	0.8132 (4)	0.0933 (3)	0.42821 (17)	0.0289 (7)	
C6	0.7662 (5)	0.0350 (4)	0.4965 (2)	0.0383 (8)	
H6A	0.7139	−0.0486	0.4953	0.046*	
C7	0.7979 (5)	0.1027 (4)	0.56667 (19)	0.0419 (9)	
H7A	0.7705	0.0639	0.6134	0.050*	
C8	0.8695 (5)	0.2266 (4)	0.56639 (19)	0.0388 (8)	
H8A	0.8870	0.2721	0.6136	0.047*	
C9	0.8878 (4)	0.2192 (3)	0.43411 (18)	0.0336 (8)	
H9A	0.9199	0.2592	0.3885	0.040*	
Cl1	0.65614 (11)	0.77575 (8)	0.69869 (5)	0.0395 (3)	
O2	0.6939 (3)	0.9043 (3)	0.73148 (17)	0.0567 (7)	
O1	0.5062 (4)	0.7213 (3)	0.7307 (2)	0.0798 (11)	
O3	0.6227 (7)	0.7890 (4)	0.6170 (2)	0.1085 (14)	
O4	0.8049 (5)	0.6906 (3)	0.7127 (3)	0.0937 (12)	
O1W	0.7229 (3)	0.0328 (3)	−0.06446 (14)	0.0425 (6)	
H1WA	0.8172	0.0228	−0.0338	0.051*	
H1WB	0.7353	0.0972	−0.0954	0.051*	

### Atomic displacement parameters (Å^2^)

**Table:**

	*U*^11^	*U*^22^	*U*^33^	*U*^12^	*U*^13^	*U*^23^
Zn1	0.0407 (4)	0.0362 (4)	0.0182 (3)	0.0009 (2)	0.0001 (2)	0.00160 (19)
N1	0.0336 (15)	0.0363 (16)	0.0236 (14)	0.0012 (12)	−0.0006 (11)	−0.0013 (11)
N2	0.0366 (16)	0.0505 (18)	0.0295 (16)	−0.0022 (14)	0.0039 (12)	−0.0054 (13)
N3	0.0340 (14)	0.0331 (15)	0.0205 (13)	−0.0001 (12)	−0.0014 (11)	−0.0046 (11)
N4	0.0432 (16)	0.0377 (16)	0.0202 (14)	−0.0052 (13)	−0.0014 (11)	−0.0075 (11)
N5	0.0452 (17)	0.0345 (16)	0.0235 (14)	−0.0029 (13)	−0.0003 (11)	−0.0017 (11)
C1	0.0358 (18)	0.0342 (18)	0.0231 (17)	0.0003 (14)	0.0018 (14)	−0.0037 (13)
C2	0.0329 (18)	0.049 (2)	0.0310 (19)	−0.0013 (16)	0.0051 (14)	−0.0044 (16)
C3	0.0302 (17)	0.0324 (18)	0.0281 (17)	0.0047 (14)	−0.0034 (14)	−0.0024 (14)
C4	0.060 (2)	0.035 (2)	0.0332 (19)	−0.0104 (17)	−0.0008 (16)	−0.0012 (15)
C5	0.0290 (16)	0.0318 (18)	0.0255 (16)	0.0025 (13)	0.0006 (12)	0.0020 (13)
C6	0.042 (2)	0.0398 (19)	0.0335 (19)	−0.0050 (16)	0.0045 (15)	0.0020 (15)
C7	0.051 (2)	0.048 (2)	0.0270 (18)	−0.0056 (17)	0.0055 (15)	0.0059 (15)
C8	0.050 (2)	0.044 (2)	0.0222 (17)	−0.0012 (16)	0.0023 (15)	−0.0005 (14)
C9	0.0439 (19)	0.0359 (19)	0.0212 (16)	−0.0003 (15)	0.0035 (13)	0.0014 (13)
Cl1	0.0451 (5)	0.0349 (5)	0.0397 (5)	0.0018 (4)	0.0111 (4)	−0.0036 (3)
O2	0.0545 (16)	0.0439 (16)	0.073 (2)	−0.0038 (13)	0.0105 (14)	−0.0182 (13)
O1	0.068 (2)	0.067 (2)	0.110 (3)	−0.0251 (17)	0.043 (2)	−0.0256 (18)
O3	0.196 (4)	0.089 (3)	0.041 (2)	0.001 (3)	0.010 (2)	−0.0046 (18)
O4	0.067 (2)	0.054 (2)	0.161 (4)	0.0276 (17)	0.012 (2)	0.011 (2)
O1W	0.0432 (14)	0.0514 (16)	0.0339 (14)	−0.0006 (12)	0.0092 (11)	0.0095 (11)

### Geometric parameters (Å, °)

**Table:**

Zn1—O1W	2.106 (2)	C3—C5	1.491 (4)
Zn1—O1W^i^	2.106 (2)	C4—H4B	0.9600
Zn1—N1^i^	2.111 (3)	C4—H4C	0.9600
Zn1—N1	2.111 (3)	C4—H4D	0.9600
Zn1—N5^ii^	2.245 (3)	C5—C6	1.382 (4)
Zn1—N5^iii^	2.245 (3)	C5—C9	1.389 (5)
N1—C1	1.301 (4)	C6—C7	1.387 (5)
N1—N2	1.385 (4)	C6—H6A	0.9300
N2—C2	1.292 (4)	C7—C8	1.362 (5)
N3—C1	1.352 (4)	C7—H7A	0.9300
N3—C2	1.354 (4)	C8—H8A	0.9300
N3—N4	1.408 (3)	C9—H9A	0.9300
N4—C3	1.276 (4)	Cl1—O1	1.404 (3)
N5—C9	1.332 (4)	Cl1—O3	1.409 (4)
N5—C8	1.349 (4)	Cl1—O4	1.412 (3)
N5—Zn1^iv^	2.245 (3)	Cl1—O2	1.433 (3)
C1—H1B	0.9300	O1W—H1WA	0.8500
C2—H2B	0.9300	O1W—H1WB	0.8500
C3—C4	1.491 (5)		
			
O1W—Zn1—O1W^i^	180	N4—C3—C5	112.9 (3)
O1W—Zn1—N1^i^	92.38 (10)	C4—C3—C5	120.0 (3)
O1W^i^—Zn1—N1^i^	87.62 (10)	C3—C4—H4B	109.5
O1W—Zn1—N1	87.62 (10)	C3—C4—H4C	109.5
O1W^i^—Zn1—N1	92.38 (10)	H4B—C4—H4C	109.5
N1^i^—Zn1—N1	180	C3—C4—H4D	109.5
O1W—Zn1—N5^ii^	94.97 (10)	H4B—C4—H4D	109.5
O1W^i^—Zn1—N5^ii^	85.03 (10)	H4C—C4—H4D	109.5
N1^i^—Zn1—N5^ii^	87.73 (10)	C6—C5—C9	117.2 (3)
N1—Zn1—N5^ii^	92.27 (10)	C6—C5—C3	123.4 (3)
O1W—Zn1—N5^iii^	85.03 (10)	C9—C5—C3	119.3 (3)
O1W^i^—Zn1—N5^iii^	94.97 (10)	C5—C6—C7	119.2 (3)
N1^i^—Zn1—N5^iii^	92.27 (10)	C5—C6—H6A	120.4
N1—Zn1—N5^iii^	87.73 (10)	C7—C6—H6A	120.4
N5^ii^—Zn1—N5^iii^	180	C8—C7—C6	119.2 (3)
C1—N1—N2	108.4 (3)	C8—C7—H7A	120.4
C1—N1—Zn1	133.6 (2)	C6—C7—H7A	120.4
N2—N1—Zn1	117.9 (2)	N5—C8—C7	123.0 (3)
C2—N2—N1	106.1 (3)	N5—C8—H8A	118.5
C1—N3—C2	105.5 (3)	C7—C8—H8A	118.5
C1—N3—N4	129.8 (3)	N5—C9—C5	124.3 (3)
C2—N3—N4	122.9 (3)	N5—C9—H9A	117.8
C3—N4—N3	118.2 (3)	C5—C9—H9A	117.8
C9—N5—C8	116.9 (3)	O1—Cl1—O3	110.2 (3)
C9—N5—Zn1^iv^	120.6 (2)	O1—Cl1—O4	110.0 (2)
C8—N5—Zn1^iv^	121.9 (2)	O3—Cl1—O4	107.3 (3)
N1—C1—N3	109.0 (3)	O1—Cl1—O2	109.83 (17)
N1—C1—H1B	125.5	O3—Cl1—O2	108.5 (2)
N3—C1—H1B	125.5	O4—Cl1—O2	111.0 (2)
N2—C2—N3	110.9 (3)	Zn1—O1W—H1WA	108.1
N2—C2—H2B	124.5	Zn1—O1W—H1WB	125.6
N3—C2—H2B	124.5	H1WA—O1W—H1WB	110.4
N4—C3—C4	127.1 (3)		

Symmetry codes: (i) −*x*+1, −*y*, −*z*; (ii) *x*−1/2, −*y*+1/2, *z*−1/2; (iii) −*x*+3/2, *y*−1/2, −*z*+1/2; (iv) −*x*+3/2, *y*+1/2, −*z*+1/2.

### Hydrogen-bond geometry (Å, °)

**Table:**

*D*—H···*A*	*D*—H	H···*A*	*D*···*A*	*D*—H···*A*
O1W—H1WA···N2	0.85	2.20	2.814 (4)	130
O1W—H1WB···O4^iii^	0.85	2.22	2.993 (5)	151
O1W—H1WB···O3^iii^	0.85	2.26	3.003 (5)	147

Symmetry codes: (iii) −*x*+3/2, *y*−1/2, −*z*+1/2.
